# The Use of a Fractional Laser in Acne Scar Treatment—A Systematic Review

**DOI:** 10.3390/life15060915

**Published:** 2025-06-04

**Authors:** Bartłomiej Ptaszek, Marzena Czernecka, Szymon Podsiadło

**Affiliations:** 1 Institute of Applied Sciences, University of Physical Culture in Krakow, 31-571 Krakow, Poland; 2 Faculty of Motor Rehabilitation, University of Physical Culture in Krakow, 31-571 Krakow, Poland; 3 Institute of Clinical Rehabilitation, University of Physical Culture in Krakow, 31-571 Krakow, Poland

**Keywords:** fractional laser, laser CO_2_, acne scars, therapy

## Abstract

**Background/Objectives:** Acne scars are an unwanted reminder of past acne. They constitute a significant esthetic and psychosocial problem, negatively affecting the quality of life of patients. There are many methods used to treat acne scars. One of them is fractional CO_2_ laser treatment, which stimulates the skin to produce collagen. The main aim of this study was to verify, based on the available literature, the effectiveness of fractional CO_2_ laser treatment of acne scars and to determine the potential risk associated with the use of this therapeutic method. **Methods:** The literature review includes English-language articles selected using keywords and inclusion and exclusion criteria. In order to select appropriate sources, databases such as PubMed and Google Scholar were searched. **Results:** The systematic review included seven studies that assessed the effectiveness of fractional CO_2_ laser therapy for acne scars. Most patients experienced a 30% to 70% improvement in the appearance of their scars. The most commonly reported adverse events during treatment were transient erythema, edema, and post-inflammatory hyperpigmentation, which resolved within a few weeks. **Conclusions:** Fractional CO_2_ laser therapy is an effective and safe treatment for acne scars. However, despite the abundance of evidence, there is a need for further studies focusing on long-term monitoring of patients.

## 1. Introduction

Acne vulgaris is one of the most common skin dermatoses, affecting both adolescents and adults. Despite progress in treatment, it often leaves behind a variety of marks and scars that can persist for many years [1]. These changes constitute a significant esthetic and psychosocial problem, negatively affecting the quality of life. These defects can lead to lower self-esteem, problems with self-confidence and difficulties in social interactions. They are often accompanied by feelings of shame and uncertainty, which may require psychological intervention [2].

Acne scars are the result of skin healing processes. The changes that appear in the course of acne, especially inflammatory and purulent conditions, activate skin repair processes [1,3]. Acne scars are diverse in terms of appearance, depth and the mechanism of formation. There are two main types of scars: atrophic, sunken below the skin level (ice-pick, boxcar, rolling), and hypertrophic, raised above the skin level (pink or red changes appear as a result of an abnormal wound healing process, they do not extend beyond the wound borders) [1,3,4]. Scars are a natural result of the wound healing process, but their final appearance depends on many factors. Both general and local factors have an impact on this. The first group includes age, skin color, the general condition of the body, diabetes, kidney and liver diseases, hormonal disorders, nutrition, genes and individual predispositions to the formation of hypertrophic and keloid scars. Local factors include location, the width and shape of the wound, abnormal blood supply to the wound, infections at the site of scar formation, and wound care [1].

Acne scar treatment has been a significant problem for both cosmetologists and dermatologists for many years. Currently, there are many treatment methods available, but they are not always effective. It is important to choose the right method for the severity of the scars and the type of skin [3,5].

The fractional CO_2_ laser (carbon dioxide) was built in the 1960s. It has been used in medicine for many years, especially in dermatology and cosmetology. The active medium of this type of laser is a mixture of gases such as carbon dioxide, nitrogen, hydrogen and helium. The CO_2_ laser emits infrared waves with a wavelength of 10,600 nm. It works by creating regularly spaced, vertical micro-damages in the skin. Light beams reaching deep into the skin are absorbed by the water contained in the tissues, resulting in a photothermal reaction. Heating the water in the tissues leads to its evaporation and the release of heat, which damages the surrounding cells of the body. The effect of the damage is the initiation of skin repair processes, which results in the formation of new collagen fibers. As a result, the skin becomes thicker, tighter and more elastic [6,7,8].

The main aim of the study was to verify, based on the available literature, the effectiveness of acne scar treatment with a fractional CO_2_ laser and to determine the potential risk associated with the use of this therapeutic method.

## 2. Materials and Methods

In this paper, a systematic review of scientific articles was conducted based on online databases such as PubMed and Google Scholar. The research material consisted of English-language papers from the last 14 years. The search was conducted using keywords: fractional laser, CO_2_ laser, scars, acne scars, therapy.

**Figure 1:**
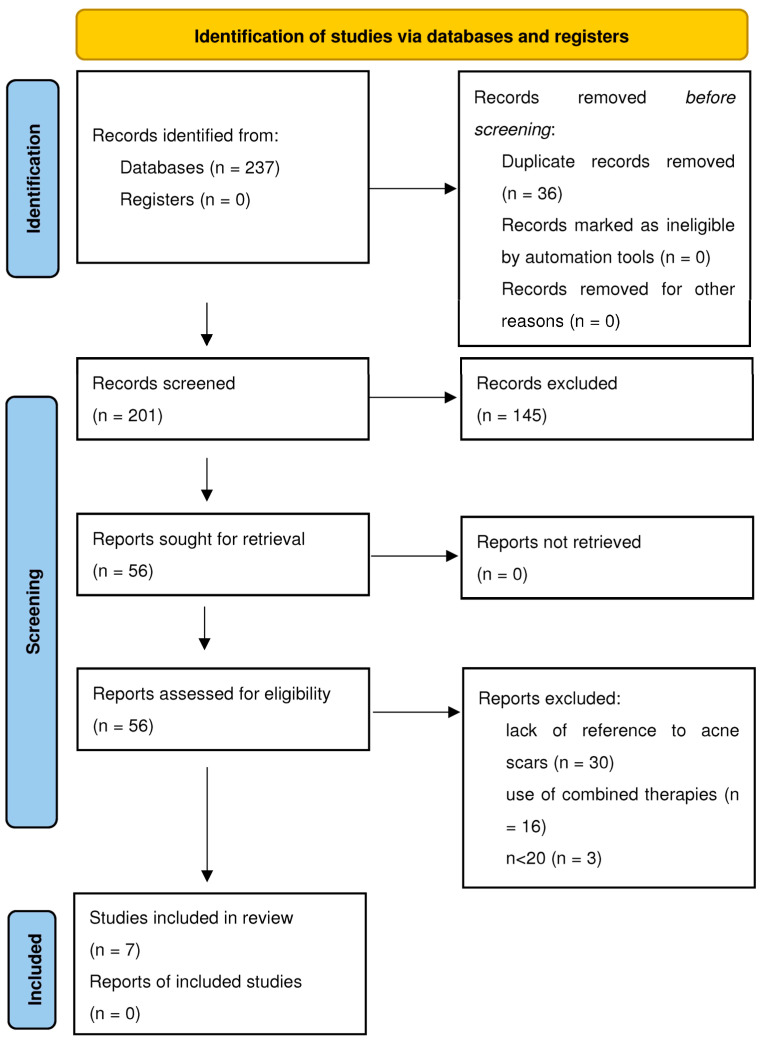
PRISMA flow diagram.

Search results (PubMed n = 140, Google Scholar n = 97, total n = 237) → After removing duplicates (n = 201) → After rejection based on abstracts (n = 56) → After rejection due to lack of reference to acne scars, use of combined therapies or n < 20 (n = 7) (Figure 1).

## 3. Results

The results of the review are presented in Table 1, Table 2, Table 3, Table 4 and Table 5.

## 4. Discussion

Acne scars are an unwanted reminder of past acne. They are caused by the improper healing of inflammation. Treating them is difficult and challenging for cosmetologists and dermatologists [1]. Fractional CO_2_ laser treatments have become an increasingly popular method of treating acne scars over the years. Many people decide on this form of therapy due to its high effectiveness and low invasiveness. During the treatment, controlled cell damage occurs, which leads to the initiation of skin repair processes and the creation of new collagen fibers [7]. The results of the discussed studies show that fractional laser therapies bring satisfactory therapeutic effects. In all study groups, mild to moderate improvement was observed (30–70% improvement in the appearance of scars). In the study conducted by Ahmad et al. (2012), scars almost completely disappeared in two patients [9]. No worsening of the condition was noted in any study. Most of the studies conducted referred to short-term effects of the therapy. In two cases, the results were assessed three months after the end of the treatment, while in three cases they were assessed after six months. In the study by Qian et al. (2012) [15], the results of the therapy were also assessed after twelve months. The effects of the therapy after one year were usually better than after three months [15]. In the study conducted by Elcin et al. (2017) [11], the assessment was also performed after three years. The long-term follow-up period allowed for recording a statistically significant difference in the improvement of the appearance of scars [11]. The study conducted by Walgrave et al. (2009) [16] also confirmed the effectiveness of fractional laser therapy. During the therapy, one to three treatments were performed, and the assessment was performed three months after the last treatment. In most patients, mild or moderate improvement in skin texture and scar appearance was observed [16].

It is worth noting that despite the relative safety of fractional CO_2_ laser treatments, post-treatment complications still occur. According to the literature review, the most common side effects were erythema and swelling lasting up to several weeks after the procedure. However, the occurrence of adverse effects is unavoidable due to the increased blood flow that accompanies the inflammatory reaction induced by the laser [17]. During the procedure, patients also reported mild pain, burning or itching that lasted up to several hours. Other complications that were noted were pinpoint bleeding, transient post-inflammatory discoloration/hyperpigmentation, exfoliation and scab formation. Additionally, in the study conducted by Elcin et al. (2017) [11], six patients experienced hyperpigmentation lasting longer than a month, while four experienced acne exacerbation. All patients who experienced long-term complications required treatment [11]. However, proper preparation of the patient for the procedure, as well as adherence to post-procedure recommendations, can significantly reduce the risk of adverse effects. Another important aspect is the selection of appropriate treatment parameters for the type of scars and the patient’s skin.

### 4.1. Fractional CO_2_ Laser vs. Chemical Peeling

CO_2_ laser therapy is one of the most effective methods used to reduce acne scars. However, due to the recovery time and potential side effects, some people choose less-invasive methods that are less invasive to the tissue, but may require more treatments or be less effective in some cases. A study conducted by Ahmed et al. (2014) [18] on 28 participants with ice-pick scars compared the effectiveness of fractional CO_2_ laser therapy with 100% TCA (trichloroacetic acid). The subjects were randomly assigned to two groups. Four treatments were performed at three-week intervals. Both the laser and TCA were applied locally to reach the bottom of the scar. After completing the therapy, there was a significant improvement in the appearance and reduction of scars in the fractional CO_2_ laser group compared to the trichloroacetic acid group. In the CO_2_ laser group, four patients experienced adverse events such as transient post-inflammatory hyperpigmentation or the appearance of pustules. In the second group, all patients experienced complications such as itching, infection, and transient post-inflammatory hyperpigmentation [18].

### 4.2. Combined Fractional CO_2_ Laser Therapy with Other Methods

Combined therapies using different treatment methods are very often used in cosmetology to achieve better results. Combining a fractional CO_2_ laser with other treatments can increase the effectiveness of acne scar treatment or shorten the recovery time. A study conducted by Faghihi et al. (2016) [19], which involved sixteen participants, tested the effectiveness of autologous platelet-rich plasma in combination with a fractional CO_2_ laser. Patients received two treatments at monthly intervals. One half of the face underwent CO_2_ laser treatment in combination with intradermal administration of platelet-rich plasma, while the other half underwent CO_2_ laser treatment in combination with intradermal administration of saline. After the end of the therapy, greater improvement was noted on the side treated with the CO_2_ laser in combination with platelet-rich plasma than on the opposite side. However, this difference was not statistically significant. During the therapy, it was noted that the side additionally treated with platelet-rich plasma had significantly more severe erythema and swelling, lasting for a longer period of time, than the opposite side [19].

A similar study was conducted by Kar et al. (2017) [20] in thirty patients who underwent three treatments at monthly intervals. A fractional CO_2_ laser combined with intradermal platelet-rich plasma was used on one half of the face, while CO_2_ laser treatment alone was performed on the other half. Improvement occurred on both sides of the face, and the difference between them was not statistically significant. However, in contrast to the previously described study, redness and swelling were lower on the side additionally treated with platelet-rich plasma [20]. The differences in the results of both studies mean that more clinical trials are needed to test the efficacy and safety of fractional CO_2_ laser therapy combined with platelet-rich plasma.

Microneedle radiofrequency is a procedure that combines microneedling with the delivery of thermal energy into the skin. The study by Kim et al. (2023) [21] compared the efficacy of fractional laser therapy and the same laser combined with microneedle radiofrequency. The therapy was performed on 23 people who underwent three treatments at 4-week intervals. Half of the face received laser therapy alone, while the other half received combined therapy. There was no difference in the frequency of side effects, such as redness and swelling, on both sides of the face [21].

Kim (2008) [22] conducted a study involving thirty-five patients with moderate to severe acne scars. The treatments were repeated a maximum of five times at two- to three-week intervals. During the treatments, spot irradiation with a fractional laser and puncturing of the previously irradiated scar area were performed. The puncturing technique consisted of 5–10 punctures with a needle to a depth of about 1 mm. As a result, all patients showed improvement. Spot irradiation resulted in minimal side effects and a shortened recovery time [22].

Mohammed (2013) [23] used the Kim (2008) [22] method in her study involving sixty patients. The subjects were divided into two groups. The first group received CO_2_ laser treatments only, while the second group received additional puncturing with a needle. Improvement in scar appearance was observed in all subjects, but there was no statistically significant difference in the results between the two groups. Reported side effects were minimal, including mild erythema, swelling, and minor crusting [23]. Combination therapies may be more effective than monotherapies. However, further clinical trials seem necessary to refine treatment processes and investigate the efficacy and safety of specific therapies.

### 4.3. Summary

A review of the literature has shown that a fractional CO_2_ laser is an effective method of treating acne scars. This is confirmed by numerous clinical studies. Most studies show significant improvement in the appearance of scars after just a few treatments, which is confirmed by dermatologists’ assessments and the subjective feelings of patients. Scar reduction was most often assessed at a level of 30% to 70%. Such results are satisfactory for both patients and therapists. It is worth noting, however, that the effects of the therapy depend on many factors. One of the most important factors influencing the effectiveness of the therapy is the depth of acne scars. In the case of deep scars, it may be necessary to carry out more treatments to achieve satisfactory results. Another important aspect is the selection of appropriate laser parameters during the treatments. Energy, pulse density and the number of passes performed on a given area of the skin are crucial to achieving optimal results. Although fractional CO_2_ laser therapy is generally considered to be safe, a review of the literature shows that certain side effects may occur, affecting patient comfort and recovery time. The most common complications are erythema, swelling and post-inflammatory discoloration, which usually disappear spontaneously within a few weeks. It is also worth noting that despite numerous studies confirming the effectiveness of fractional CO_2_ laser therapy, there are several limitations that should be taken into account when interpreting the results. Most clinical studies include relatively small study groups, which may make it difficult to generalize the results. Another important aspect is the small number of studies focusing on the long-term assessment of post-treatment effects. Usually, short-term results are assessed (up to six months after the end of therapy), which does not allow for full verification of how long the effects last and what the possible long-term complications are. In addition, the variability in the assessment of the effectiveness of the therapy, for example, the use of different scar assessment scales, may make it difficult to compare results between studies. Considering the above limitations, further studies should focus on conducting the therapy with a larger number of patients and long-term follow-up to better assess the durability of the effects and possible complications.

## 5. Conclusions

Fractional CO_2_ laser acne scar treatment is an effective treatment method, but its effectiveness depends on several factors, such as the type, depth of scars and treatment parameters.

Fractional CO_2_ laser acne scar treatment is generally considered to be a safe treatment method, but there is a risk of side effects, such as erythema, swelling, post-inflammatory discoloration, and pinpoint bleeding, which may affect the final result of the therapy.

## Figures and Tables

**Table 1:** Summary of data on the subjects examined.

Author of the Study	Number of Respondents	Sex	Age
Ahmad et al. [9]	20	17–W, 3–M	21–34
Asilian et al. [10]	32	22–W, 10–M	19–43
Elcin et al. [11]	31	15–W, 16–M	18–59
Hsiao et al. [12]	25	6–W, 19–M	19–39
Huang [13]	44	31–W, 13–M	18–37
Manuskiatti et al. [14]	24	not specified	22–51
Qian et al. [15]	31	11–W, 20–M	16–28

**Table 2:** Research inclusion and exclusion criteria.

Author of the Study	Inclusion Criteria	Exclusion Criteria
Ahmad et al. [9]	male or female gender presence of mild to moderate atrophic scars written consent to perform the test	previous treatments such as chemical peeling or dermabrasion use of oral or topical retinoids
Asilian et al. [10]	presence of any type of atrophic scarring ranging from moderate to severe	pregnancy breastfeeding tendency to develop keloid scars use of immunosuppressive drugs, isotretinoin previously performed treatments, such as: fillers, dermabrasion, laser therapy
Elcin et al. [11]	18 ears of age presence of atrophic scars on the face skin phototype I, II or III written consent to perform the test	pregnancy, lactation hypertrophic or keloid scars skin phototype IV or higher active inflammation infections or malignant tumors in the treatment area procedures performed in the last six months before the examination: laser therapy, radiofrequency, phototherapy taking retinoids in the last six months before the examination
Hsiao et al. [12]	presence of acne scars in the mild to moderate stage skin phototype III or IV	pregnancy, lactation immunosuppression use of isotretinoin tendency to develop keloid scars
Huang [13]	presence of moderate to severe acne scars skin phototype IV	not specified
Manuskiatti et al. [14]	skin phototype IV shallow or deep boxcar scars that appeared at least 6 months before the examination written consent to perform the examination	pregnancy, breastfeeding tendency to develop keloid scars use of isotretinoin treatments using fillers or laser therapy within the last 12 months preceding the examination
Qian et al. [15]	skin phototype III or IV atrophic scars of the rolling, ice-pick or boxcar type	photosensitivity tendency to develop keloid scars pregnancy, breastfeeding autoimmune diseases active acne use of retinoids within the last month before entering the study treatments performed within the last six months before the study: laser skin resurfacing, dermabrasion, chemical peeling

**Table 3:** Treatment procedures.

Author of the Study	Device Name	Parameters	Treatment Series
Ahmad et al. [9]	Max 7000, Republic of Korea	10–20 mJ/cm^2^ density 2–3 deep resurfacing mode used	6 treatments every month
Asilian et al. [10]	Pixel Alma 10,600 nm, Israel	pulse duration 350 μs coagulation zone 350 μm	4 treatments every four weeks
Elcin et al. [11]	Lutronic, eCO_2_, Seoul, Republic of Korea	100–160 mJ spot diameter 120 μm spot density 75–100/cm^2^ power 30 W	2–3 treatments every four weeks
Hsiao et al. [12]	Lumenis UltraPulse Encore fractional CO_2_ laser, CA, USA	Deep FX mode: 12.5–15 mJ density 2–3 300 Hz	1 treatment
Huang [13]	Lumenis UltraPulse Encore fractional CO_2_ laser, CA, USA	Stage I—Active FX Mode: 100–150 mJ density 5–7 Stage II—Deep FX Mode: 10–15 mJ density 10–15% Stage III—Active FX Mode: 100–125 mJ density 1–2	minimum 2 treatments (2–7) within 2–6 months
Manuskiatti et al. [14]	Lumenis AcuPulse, Santa Clara, CA, USA	12.5–15 mJ pulse duration 950 μs skin coverage 5%	2 treatments every two months
Qian et al. [15]	Lumenis Ultrapulse 5000, ActiveFx, CA, USA	150–200 mJ density 5–6 spot size 1.2 mm pulse frequency 30–50 Hz	3 treatments every two months

**Table 4:** Evaluation period and research assessment tools.

Author of the Study	Result Evaluation Period	Assessment Tools
Ahmad et al. [9]	Before the series of treatments, the severity of scars was graded and photographs were taken. Assessments were made before and after each treatment. The final assessment was made six months after the last treatment.	photographs taken before and after the procedure four-point scale for assessing the improvement of acne scars
Asilian et al. [10]	Assessments were performed at the beginning of therapy and three and six months after the end of therapy.	optical imaging system—photographs taken using a Canon Power shot G12 camera four-point scale for assessing the improvement of acne scars (<25% slight improvement, 25–50% moderate improvement, 51–75% good improvement, >75% excellent improvement) assessment based on photographs was performed by two dermatologists and patients
Elcin et al. [11]	Assessments were performed before each treatment and 1, 2, 3 months, and 3 years after the completion of therapy.	photographs taken before each treatment and 1, 2, 3 months, and 3 years after the therapy was completed assessment was performed by two independent doctors using the ECCA scale four-point scale for assessing the improvement of acne scars (<25% slight improvement, 25–50% moderate improvement, 51–75% significant improvement, >75% almost complete improvement) patients assessed on a six-point scale (−1 is worsening, 0 is no change, 1 is mild improvement, 2 is moderate improvement, 3 is significant improvement, 4 is almost complete improvement)
Hsiao et al. [12]	Assessments were made at the beginning of therapy, one week, one month, and three months after its completion.	digital photographs VISIA Complexion Analysis software four-point scale for assessing the improvement of acne scars (1 ≤ 25% improvement, 2 = 26–50% improvement, 3 = 51–75% improvement, 4 ≥ 75% improvement) statistical analysis—Wilcoxon test
Huang [13]	Assessments were performed in the first week and one, two and three months after the end of laser therapy.	digital photographs four-point scale focusing on scar appearance and skin texture (≤25% mild improvement, 26–50% moderate improvement, 51–75% significant improvement, >75% excellent improvement)
Manuskiatti et al. [14]	Photographs were taken before each treatment, as well as one, three, and six months after the end of therapy. After each treatment, patients assessed their pain level.	Visioscan VC98 camera with SELS (Surface Evaluation of the Living Skin) software photographs taken before each treatment using a Canon PowerShot G9 camera two independent dermatologists made the assessment using a four-point scale: 0 ≤ 25%, 1 = 25–50%, 2 = 51–75%, 3 ≥ 75% improvement ten-point pain scale (from 0 = no pain to 10 = severe pain) assessment of the effects made by the subjects (<25% slight improvement, 25–50% fair improvement, 51–75% good improvement, >75% excellent improvement) statistical analysis—Wilcoxon test
Qian et al. [15]	The results were assessed three and twelve months after the end of therapy.	photographs taken before and after each treatment using a Canon EOS 400D digital camera two independent dermatologists made assessments using a four-point scale: poor improvement (25%), moderate improvement (26–50%), good improvement (51–75%) or excellent improvement (76–100%) statistical analysis using the chi-square test

**Table 5:** Research results.

Author of the Study	Results	Side Effects
Ahmad et al. [9]	The effects of the therapy were assessed by 16 study participants. A mild or moderate improvement was observed in most patients, while in two patients, the scars almost completely disappeared.	mild pain or burning crusting desquamation transient redness or hyperpigmentation
Asilian et al. [10]	At six months after completing treatment, most patients (84.4%) reported moderate or good improvement in the appearance of their scars. Only one patient (3.1%) rated the response to treatment as excellent, while four patients (12.5%) rated it as mild improvement. The ratings given by two independent dermatologists differed slightly from those given by the patients. In this case, mild improvement was noted in 18.8%, moderate or good in 78.8%, and excellent in 3.1%.	burning sensation up to several hours after the procedure temporary post-inflammatory discoloration
Elcin et al. [11]	Short-term follow-up (3 months after completion of therapy) When evaluating the results based on the ECCA scale, a statistically significant decrease in the mean number of points indicating improvement was noted. No change was noted in 7 people, while the remaining people showed mild to moderate improvement. None of the patients experienced worsening or almost complete improvement. Assessment by the study participants After three months from completion of therapy, most patients reported mild to almost complete improvement. Two participants reported worsening and four reported no change. Long-term follow-up (3 years after completion of therapy) Six patients agreed to re-evaluation. A statistically significant difference in the improvement of scar appearance was noted.	transient erythema and swelling pinpoint bleeding hyperpigmentation lasting more than a month, requiring treatment acne exacerbation, requiring treatment
Hsiao et al. [12]	After one month of treatment, first- or second-degree improvement was noted (mean 1.96), while after three months, greater improvement was noted (mean 2.41). In the VISIA study, after one month, skin texture and pore appearance improved, but redness increased. After three months, the improvement was stable and most of the discoloration had disappeared.	transient erythema pruritus occurrence of transient post-inflammatory discoloration
Huang [13]	Most patients showed significant improvement, three showed excellent improvement, and the remaining 13 patients showed mild or moderate improvement.	pinpoint bleeding transient swelling and erythema post-inflammatory hyperpigmentation occurrence of HSV foci—cured within seven days
Manuskiatti et al. [14]	Twenty patients completed the study (12 women, 8 men). Three months after the end of therapy, 40% of the subjects showed more than 50% improvement, and after six months, this percentage increased to 65%. The assessments made by the patients did not differ from the assessments made by the doctors. No patient showed any deterioration in the appearance of the scars. Analysis using Visioscan showed a reduction in scar volume compared to the initial assessment. After three months, the reduction in scar volume was 9.5%, and after six months it was 13.2%.	post-inflammatory hyperpigmentation lasting four to seven weeks pinpoint bleeding superficial scabs transient erythema and swelling lasting for 24 h
Qian et al. [15]	After 3 months of therapy, an excellent response to treatment was noted in 6.5% of the study subjects, good or satisfactory in 64.5%, and poor in 29%. After 12 months, 12.9% of patients noted excellent improvement, 67.7% good or satisfactory, and 19.4% poor. The response to treatment after 12 months was usually better than after 3 months, but this difference was not statistically significant.	mild, diffuse facial erythema with swelling lasting 24 to 48 h moderate to severe stinging sensation lasting up to an hour after the procedure mild bleeding for 1–2 days occurrence of transient post-inflammatory discoloration

## Data Availability

Not applicable.
